# Validation of a New Sensitive Method for the Detection and Quantification of *R* and *S*-Epimers of Ergot Alkaloids in Canadian Spring Wheat Utilizing Deuterated Lysergic Acid Diethylamide as an Internal Standard

**DOI:** 10.3390/toxins14010022

**Published:** 2021-12-31

**Authors:** Jensen Cherewyk, Taylor Grusie-Ogilvie, Barry Blakley, Ahmad Al-Dissi

**Affiliations:** 1 Department of Veterinary Biomedical Sciences, Western College of Veterinary Medicine, University of Saskatchewan, Saskatoon, SK S7N 5B4, Canada; brb237@mail.usask.ca; 2 Prairie Diagnostic Services (PDS), Saskatoon, SK S7N 5B4, Canada; taylor.ogilvie@pds.usask.ca; 3 Department of Veterinary Pathology, Western College of Veterinary Medicine, University of Saskatchewan, Saskatoon, SK S7N 5B4, Canada; ahmad.aldissi@usask.ca

**Keywords:** *Claviceps purpurea*, liquid chromatography, mass spectrometry

## Abstract

Ergot sclerotia effect cereal crops intended for consumption. Ergot alkaloids within ergot sclerotia are assessed to ensure contamination is below safety standards established for human and animal health. Ergot alkaloids exist in two configurations, the *R* and *S*-epimers. It is important to quantify both configurations. The objective of this study was to validate a new ultra-high performance liquid chromatography tandem mass spectrometry (UHPLC-MS/MS) method for quantification of six *R* and six *S*-epimers of ergot alkaloids in hard red spring wheat utilizing deuterated lysergic acid diethylamide (LSD-D_3_) as an internal standard. Validation parameters such as linearity, limit of detection (LOD), limit of quantification (LOQ), matrix effects, recovery and precision were investigated. For the 12 epimers analyzed, low LOD and LOQ values were observed, allowing for the sensitive detection of ergot epimers. Matrix effects ranged between 101–113% in a representative wheat matrix. Recovery was 68.3–119.1% with an inter-day precision of <24% relative standard deviation (RSD). The validation parameters conform with previous studies and exhibit differences between the *R* and *S*-epimers which has been rarely documented. This new sensitive method allows for the use of a new internal standard and can be incorporated and applied to research or diagnostic laboratories.

## 1. Introduction

Mycotoxins continue to be a concern for the safety and quality of food and feed [1,2]. Specifically, the fungus *Claviceps purpurea* infects cereal crops forming visibly dark sclerotia that contain secondary metabolites known as ergot alkaloids [3]. Ergot alkaloids have two configurations known as the *R*-epimer and *S*-epimer. Rotation of a functional group on the chemical structure at the carbon-8 defines the epimer. The *R*-epimer exhibits a left-hand rotation, whereas the *S*-epimer exhibits a right-hand rotation [4,5,6]. Of the ergot alkaloids produced by *Claviceps purpurea*, six *R* and six corresponding *S* epimers are quantified in food and feed samples [7]. The six *R*-epimers are, ergocornine, ergocristine, ergocryptine, ergometrine, ergosine and ergotamine [8]. The corresponding *S*-epimers are ergocorninine, ergocristinine, ergocryptinine, ergometrinine, ergosinine and ergotaminine. Ergot epimers have different concentrations within the sclerotia depending on the geographic location and crop type [9].

In North America, diagnostic laboratories routinely include *R*-epimers in their analytical methods for ergot alkaloid detection and quantification. However, the *S*-epimers are often not included in diagnostic assays or analytical studies [10,11,12]. The *S*-epimers have been considered non [13] or less [14] bioactive compared to the corresponding *R*-epimers. The *R*-epimers are known to cause adverse effects when consumed by humans and animals [15]. Similarly, the *S*-epimers may also affect physiological systems [16,17]. It is, therefore, important to quantifying *S*-epimers in ergot-contaminated samples.

Ergot alkaloids can epimerize between the *R* and *S*-epimer [18]. Komarova and Tolkachev (2001) noted that the rate of conversion may be associated with the side group of a particular epimer. The conversion, also known as epimerization, between *R* and *S*-epimers, has also been associated with pH, light, matrix, and temperature [19,20,21,22]. If *S*-epimers constitute a large portion of the total concentration of ergot epimers [3], it is imperative to include them in an analysis for regulatory or diagnostic considerations.

Multiple instruments and techniques have been used to quantify ergot alkaloids. The use of an ultra-high performance liquid chromatography tandem mass spectrometry (UHPLC-MS/MS) method has been used to quantify mycotoxins [23,24]. The UHPLC-MS/MS technique can quantify both *R* and *S*-epimers of ergot alkaloids and have become the method of choice for detection [25]. Additionally, methods such as high-performance thin-layer chromatography (HPTLC) [26], and liquid chromatography with fluorescence detection [27] have been recently utilized for the detection of the *R* and *S*-epimers of ergot alkaloids. Conversely, the use of an enzyme linked immunosorbent assay (ELISA) cannot distinguish differences among epimers [28]. The European Union recommends that both *R* and *S*-epimers of ergot alkaloids should be quantified [29]. In contrast, the Canadian Food Inspection Agency (CFIA) currently only considers the *R*-epimers in their safety standards [30].

Internal standards are regularly utilized in analytical quantification. Structural analogues, isotopic labeled, and deuterated compounds have been used as internal standards [20]. Isotopically label internal standards are ideal since they behave in a similar manner to the analytes of interest. However, such labeled internal standards are limited for all ergot epimers. Usually, one or two internal standards are used to account for losses during extraction and analysis of ergot epimers [3,27,31]. Internal standards that can successfully behave in a similar way to the analytes of interest and account for any losses of the analytes throughout extraction and analysis, are beneficial in analytical methods.

The simultaneous detection and quantification ergot *R* and *S*-epimers are beneficial in terms of time and ease of interpretation. Only low concentrations of ergot epimers in feed are accepted according to regulatory standards. Therefore, a sensitive and timely method for epimer quantification is required. Assessing similarities and differences between the *R* and *S*-epimers in terms of validation may provide useful information for future analytical methods. The objective of this study was to validate a new method for the simultaneous detection and quantification of *R* and *S*-epimers of ergot alkaloids utilizing deuterated lysergic acid diethylamide (LSD-D_3_) as a new internal standard. Deuterated lysergic acid diethylamide has not been utilized previously to quantify ergot epimers, to the authors knowledge. A second objective was to use the validated method to quantify *R* and *S*-epimers of ergot alkaloids in naturally contaminated hard red spring wheat samples.

## 2. Results

### 2.1. Method Validation

The calibration curves obtain from this method had a r^2^ > 0.99 (Table 1). The limit of detection (LOD) and limit of quantification (LOQ) were calculated according to the Guidance Document on the Estimation of LOD and LOQ for Measurements in the Field of Contaminants in Feed and Food [32]. The instrumental LOD ranged from 0.00893–0.225 µg/kg and the instrumental LOQ ranged from 0.0295–0.744 µg/kg for all 12 ergot epimers (Table 1). The LOD and LOQ, are lower for the *S*-epimers than the *R*-epimers with an exception for ergocristine/ergocristinine. The peak area (counts × minutes) for ergocorninine, ergocristinine, ergometrinine and ergotaminine (*S*) were greater than their corresponding *R*-epimers at the same concentration. Matrix effects (ME) for all 12 epimers in wheat ranged from 101–113% (Table 2). All *S*-epimers had lower matrix effects than their respective *R*-epimers. For recovery, the low concentration spike (0.75 µg/kg) had a recovery of 78.8–115.3% and the mid concentration spike (5 µg/kg) had a recovery of 68.3–119.1% (Table 2). Recovery was greater for all *S*-epimers, compared to their *R*-epimers, except for ergometrine/ergometrinine. Inter-day precision for both spiked concentrations for all epimers was <24% RSD (relative standard deviation) and intra-day precision had a RSD of <14% (Table 2). The usefulness of LSD-D_3_ as an internal standard to account for epimer losses are shown in Table 3.

### 2.2. Natural Ergot Contaminated Wheat

The total concentration of the *R* and *S*-epimers in six independent naturally contaminated hard red spring wheat samples ranged from 756 µg/kg to 942 µg/kg (Table 4). The *R*-epimers accounted for 65% of the total concentration, whereas the *S*-epimers accounted for 35%. Ergocristine had the highest concentration of all the *R*-epimers, and ergocristinine had the highest concentration of all the *S*-epimers (Table 5) and the second highest concentration of all the epimers evaluated. All concentrations were corrected by a dilution factor.

## 3. Discussion

This study describes a new and validated sensitive method for the analysis of *R* and *S*-epimers of ergot alkaloids. The validation of this method followed the validation procedures outline in the Commission Decision 2002/657/EC [33]. Various parameters were adapted from the ThermoFisher Scientific method [34], however, improved sensitivity and matrix effects are observed in the present study. The improvements could be associated with differences in the methods and/or the use of an IS. During the method validation, a limited quantity of ergot epimer standards were available to use based on cost. A lesser amount of grain and solvent were utilized to minimize cost and excessive use of standards. This allows laboratories to save money and resources, especially when sample replication is necessary and spiking at the beginning of the extraction process is important.

The linearity of r^2^ > 0.99 for the calibration curves are defined as good [29]. The LOD and LOQ were calculated using the ‘Guidance Document on the Estimation of LOD and LOQ for Measurements in the Field of Contaminants in Feed and Food’ [32]. Similarly, Arroyo-Manzanares et al., 2018 [23] and Schummer et al., 2020 [35] used the same approach. The calculated instrumental LOD and LOQ (µg/kg) from this study are low compared to Arroyo-Manzanares et al., 2018 [23], using the same matrix. The LOD for all 12 epimers are below the lowest concentration on the linear calibration curve. Food for children and infants may contain a very low concentration of ergot alkaloids according to regulatory limits [36], therefore, a new sensitive method with low LOD’s and LOQ’s are beneficial. This sensitive analytical method can be utilized in research or diagnostic research to obtain low and actuate concentrations.

For each *R*/*S*-epimer pair for each ergot alkaloids, similar and different LOD and LOQ values are observed. That could be associated with differing functional groups for certain *R/S*-epimer pairs. Potentially, the lower LOD and LOQ for most *S*-epimers could be associated with better ionization which may be related to the greater peak area (counts × minute) observed.

A common approach to determine the LOD and LOQ of a method includes the ‘mean of 3 and 10 SD’ in samples that are uninfected [29] (p. 7037) or a signal to noise ratio of 3 and 10 [31] (p. 295). The Guidance Document mentioned above suggests that common methods to determine the LOD and LOQ should not be utilized. Pascale et al., 2019 [37] mentioned that the LOD and LOQ may vary between laboratories. Therefore, the guidance document describes an approach to measure those parameters in a way to incapsulate the whole procedure to help decrease those discrepancies. Sulyok et al., 2020 [38] noted that the LOD and LOQ do not need to be reassessed for different matrices unless there is noise at a high concentration spike level. The rationale for utilizing a representative wheat matrix in the current study is supported.

Matrix effects are commonly observed when using UHPLC-MS/MS. Matrix effects of greater than 100% can infer signal enhancement, while matrix effect of less than 100% can infer signal suppression [29,31]. Variable matrix effects are observed in multiple matrices when analyzing ergot epimers [31]. Similarly, the variability in matrix effects between studies can be associated with the differences in analytical methods and instruments. In the current study, the matrix effects appear to be minimal. This is associated with ME values for each epimer occurring around 100%. There are no guidelines on the acceptable amount of matrix effects [38]. Matrix effects can be defined as soft, moderate or strong depending on the plus minus from 100%. According to that classification, the current study observed soft enhancement (100 + 20%). Associated with reasonable matrix effects and the use of an internal standard, matrix match calibration curves were not deemed necessary. Solvent calibration curves have also been utilized in recent analytical studies assessing mycotoxins [38,39]. Likewise, injecting small quantities of a matrix can minimize the need for matrix matched calibration curves [37], which was utilized in the current method.

The *S*-epimers of all ergot alkaloids had lower matrix effects, closer to 100%, compared to the *R*-epimers. This could be associated with the differences in ionization between the two epimer configurations. A lower matrix effect for the *S*-epimers may allow for a more accurate concentration analyzed.

Recovery results in the present study are similar to other analytical methods analyzing similar analytes. The low (0.75 µg/kg) spike concentration had similar recoveries for all ergot epimers (79–115%) compared to the mid (5 µg/kg) spike concentration (68–119%). Arroyo-Manzanares et al., 2018 [23] observed similar values for percent recovery with a range of 60–89% for all ergot epimers using higher spike concentrations (10 and 150 µg/kg). Tittlemier et al., 2015 [3] also observed percent recoveries ranging from 60–132% for 10 ergot epimers. Interestingly, Tkachenko et al., 2021 [39] saw greater accuracy/recovery for the *S*-epimers compared to the *R*-epimers. Similarly, this study observed the same trend except for ergometrine/-inine. However, the recovery for ergometrinine and ergometrinine are very similar. Factors such as greater ionization or greater stability may be associated with the greater recovery of the *S*-epimers compared to the *R*-epimers.

The percent recovery observed in the current study aligns with other mycotoxin standards stated in the European Commission Regulation (2006) [40], although it is stated that recovery in terms of mycotoxins is under review [38]. Krska et al., 2008 [6] related the criteria for mycotoxins as having satisfactory values of 60–120% for recovery and a RSD < 30%. Ergot alkaloids are not explicitly defined in terms of recovery in the European Commission (EC) Regulation (2006) [40].

Precision is commonly calculated using percent relative standard deviation (% RSD). Arroyo-Manzanares et al., 2018 [23] had a RSD of 13% or lower for all mycotoxins analyzed. Likewise, Guo et al., 2016 [29] had RSD values of lower than 15%. In the current study, the intra-day precision ranged from 1.86–13.81% RSD and inter-day precision ranged from 5.88–23.9% RSD for both spike concentrations. Diana Di Mavungu et al., 2012 [31] had similar results for repeatability (12–26% RSD) and within laboratory reproducibility (12–24% RSD) for the lowest spiked concentration used analyzing 12 ergot epimers. A RSD of less than 20% conforms with the European Commission Regulation (2006) [40]. An acceptable RSD ≤ 20% for 97% of the analytes quantified in different food matrices has been reported [41]. In the current study, only one analyte, ergometrine, had >20% RSD for inter-day precision. There appeared to be no trends in the similarity or differences between the *R* and *S*-epimers in terms of precision.

Internal standards are used in analytical methods to account for the loss of analytes throughout the extraction process and analytical procedure. Internal standards account for losses associated with the cleanup and detection of ergot alkaloids [20]. Ideally, for each ergot epimer analyzed, there would be an isotopically labeled internal standard. However, such internal standards are not available [36]. Therefore, in the current study, the internal standard, LSD-D_3_, was used to account for any potential losses throughout the extraction procedure and analysis. Without the internal standard, a decrease in concentration was observed which was deemed inaccurate. The observed decrease in concentration observed was associated with the dry down of the extraction solvent. Through recovery assessment, it was deemed that the use of the internal standard accounted sufficiently for the loss of all the epimers. The concentration of epimers with the correction of the internal standard were close to the actual concentration spiked. Therefore, the internal standard can be added before the dry down step to account for any potential losses. Tittlemier et al., 2015 [3] and Fabregat-Cabello et al., 2016 [42] added IS to their samples in a similar manner. The LSD-D_3_ has similar physiochemical properties to the ergot epimers, similar shape to the ergoline ring of the ergot epimers and elutes on the chromatogram similar to some epimers. A limitation of LSD-D_3_ is the deuterated atoms are cleaved during fragmentation [20]. However, the molecular ion with the deuterated atoms still attached is used for quantification. Additionally, deuterated compounds must not to have the same molecular weight as naturally occurring isotopes. Three or more deuterated atoms are recommended. The current internal standard utilized may not be ideal for all epimers analyzed compared to C13 isotopically labeled internal standard, however, it acts sufficiently for accounting for any losses. Holderied et al., 2019 used lysergic acid diethylamide (LSD) as their internal standard to detect and quantify ergot epimers associated with structural and chemical similarities. Conveniently, LSD-D_3_ can be easily purchased and is readily available, whereas LSD is a control substance in Canada. The LSD-D_3_ can now be included as an internal standard to quantify ergot alkaloids and may be adopted into current or future methods.

Epimerization of ergot epimers in analytical methods can be of concern. Cool temperature autosamplers are recommended to minimize epimerization [31,43], which was utilized in the present study. Epimerization was also minimized using amber vials and black plastic bags to limit light exposure throughout sample handling and extraction. The ultra-high performance liquid chromatography (UHPLC) column was maintain at a temperature of 40 °C. A previous method also utilized the same temperature for the quantification of ergot alkaloids [34]. As noted in the chromatogram (see materials and methods), the epimers have sharp peaks and, with the exception of ergotamine/ergotaminine, do not have a ‘saddle’ between their peaks, indicating on column epimerization is unlikely [44].

This current validated method quantified 12 ergot epimers in naturally contaminated wheat. These 12 epimers are the major ergot alkaloids produced by *Claviceps purpurea* and constitute a large portion of the ergot alkaloid metabolome [45]. Wheat samples from western Canada have had similar concentrations to the present study [3]. The *R*-epimers of each ergot alkaloid had greater concentrations than the *S*-epimers. However, the concentrations of some *R*/*S*-epimer pairs were similar to each other. Ergocristine and ergocristinine had the greatest concentrations of all *R* and *S*-epimers analyzed. Similarity, they have been reported to be the most dominate *R* and *S*-epimer in terms of concentration [19]. Interestingly, independent sample number 4 had low concentrations of ergocornine/ergocorninine compared to the other independent samples. The high proportion of *S*-epimers in contaminated grain may be contributed to the epimerization of *R* to *S*-epimers over time. Consequently, the large concentration of *S*-epimers supports the quantification in a diagnostic and analytical setting. Similarly, including the *S*-epimers of ergot alkaloids in food and feed safety standards is important.

## 4. Conclusions

This new sensitive validated UHPLC-MS/MS analytical method, through evaluation and comparison of several parameters, successfully assesses both *R* and *S* ergot epimers of ergot alkaloids. The use of LSD-D_3_ can be successfully utilized as an internal standard for the quantification of both *R* and *S*-epimers. The *R* and *S*-epimers of ergot alkaloids behave differently in this analytical method. The *S*-epimers have lower LOD and LOQ, a greater peak area, lower matrix effects, and a greater recovery than the *R*-epimers. The differences between the *R* and *S*-epimers may help advance future analytical research on ergot epimers. The high sensitivity of this method to detect and quantify ergot epimers supports the use of this method in analytical research and diagnostic settings. Especially since low concentrations of ergot alkaloids are used in regulatory standards [36]. Screening both *R* and *S*-epimers, is important because they pose a risk to the health of human and animals that consume contaminated food and feed which is currently a public health concern in developing countries [46] and in livestock feed [35]. Naturally ergot-contaminated hard red spring wheat was chosen as a representative matrix for the validation of this analytical method because it is routinely analyzed in diagnostic laboratories and multiple independent samples were readily available. Future studies will focus on validating the above method for the analysis and quantification of other matrices. This method can be used to quantify *R* and *S*-epimers of ergot alkaloids in spiked, and widely variable natural contaminated wheat samples, that are typical of a research and diagnostic setting.

## 5. Materials and Methods

### 5.1. Sample Preparation, Extraction, and Analysis

#### 5.1.1. Standards

Standards of six ergot *R*-epimers (ergotamine (95.1 ± 4.9% purity), ergometrine (98 ± 2.0% purity), ergocristine (98.7 ± 1.3% purity), ergocryptine (99.6 ± 0.4% purity), ergocornine (97.8 ± 2.2%), ergosine (95.9 ± 4.1%) and six ergot *S*-epimers (ergotaminine (95.8 ± 4.2% purity), ergometrinine (98.0 ± 2.0% purity), ergocristinine (96.6 ± 3.4% purity), ergocryptinine (99.2 ± 0.8%), ergocorninine (95.6 ± 4.4%), ergosinine (99.0 ± 1.0%) were purchased from Romer Labs (Tulln, Austria). Deuterated lysergic acid diethylamide (LSD-D_3_) was purchased from Sigma Aldrich (Oakville, ON, Canada) and used as an internal standard. Each dried standard was reconstituted in liquid chromatography-mass spectrometry (LC-MS) grade acetonitrile (≥99.9% purity) (Fisher Scientific, Edmonton, AB, Canada) to obtain concentrations of 100 µg/kg and 25 µg/kg for *R* and *S*-epimers, respectively. A working standard was made by mixing aliquots from each epimer in a high-performance liquid chromatography (HPLC) amber vial (Agilent, Santa Clara, CA, USA), with a final concentration of 312.5 µg/mL for each epimer. The working standard was dried down with nitrogen using a multivap nitrogen evaporator (Organomation, Berlin, MA, USA), capped, and stored at −80 °C until use.

#### 5.1.2. Sample Preparation

Six independent samples of ergot-contaminated hard red spring wheat were obtained from the Canadian Feed Research Centre (North Battleford, SK, Canada). Visually, each sample contained a high quantity of ergot sclerotia. Therefore, the concentration of the ergot alkaloids within each of the contaminated samples was obtained from a previous study. A serial dilution was conducted to obtain a starting concentration of ergot alkaloids that is within the proposed linear range and is relevant to ergot contamination detected routinely in feed and food samples. This was executed, instead of diluting the samples with solvent in subsequent steps, to obtain samples with more practical epimer concentrations that are observed in feed samples submitted to diagnostic labs for testing. Visually, the contaminated samples had a homogenous mixture in terms of ergot sclerotia and grain kernels. A 10 g portion of each contaminated sample was mixed with 100 g of clean hard red spring wheat by hand, whisking until a homogenous mixture was observed, with a final weight of 110 g. A Sartorius BP2100 scale (Elk Grove, CA, USA) was utilized to weigh the grain and for subsequent weighing measures. The mixture was ground using a UDY Cyclone Sample Mill (Fort Collins, CO, USA, Model #3010-060, 1 mm mesh) and mixed by hand to ensure homogeneity. Four to six grams of the mixed ground sample (amount depended on the starting concentration of the sample) was further diluted into a final weight of 404–406 g using ground clean hard red spring wheat. The final diluted sample was mixed by hand with a whisk until homogenous and was utilized for subsequent extraction and analysis.

#### 5.1.3. Extraction

The extraction procedure followed a similar approach to ThermoFisher Scientific [34], with modifications. A 5 g ground sample of the final dilution for each ergot contaminated sample was placed in a 50 mL plastic trace metal free centrifuge tube (Labconco, Kansas City, MO, USA). An extraction solvent containing 20 mL of acetonitrile:water (80:20) was added to the tube and mixed (Benchmixer Multi-tube Vortexer, Sayreville, MA, USA) for one hour. The sample was centrifuged (Beckman GPR, Indianapolis, IN, USA) for 10 min at 3500 rpm. One mL of supernatant was filtered through a 0.45 µm polytetrafluoroethylene (PTFE) syringe filter (Fisher Scientific, Edmonton, AB, Canada) into a 12 × 75 mm glass culture tube. An aliquot of 160 µL of the filtered sample was pipetted into a HPLC amber vial (Agilent, Santa Clara, CA, USA) plus 40 µL of internal standard at a concentration of 20 µg/kg. The sample was vortexed and dry down with a gentle stream of nitrogen using the multivap nitrogen evaporator at approximately 20 °C. Samples were reconstituted in 200 µL of LC-MS grade methanol (≥99.9% purity): water (50:50) (Fisher Scientific, Edmonton, AB, Canada), capped and vortexed for approximately 15 sec. A 200 µL volume of the reconstituted sample was transferred to a HPLC amber vial with 200 µL spring HPLC vial inserts (MicroSol, Eatontown, NJ, USA) and capped. The vial was centrifuged at 3500 rpm for 10 min prior to UHPLC-MS/MS analysis.

#### 5.1.4. UHPLC-MS/MS Operation and Analysis

The operation and analysis utilized some parameters outlined in ThermosFisher Scientific [34], with modifications. Separation of the ergot epimers was conducted using a ThermoFisher Scientific Vanquish UHPLC with a 2.1 mm ID filter cartridge and a Hypersil GOLD 100 mm × 2.1 mm × 1.9µm C18 Selectivity column (ThermoFisher Scientific, Waltham, MA, USA). The mobile phases consisted of LC-MS grade water (mobile phase A) and methanol (mobile phase B) each with 0.1% LC-MS grade formic acid added (Fisher Scientific, Edmonton, AB, Canada). The percent of the organic phase (mobile phase B) was modified throughout the analytical run according to the following specification: 0 min, 5%; 0.5 min, 5%; 7 min, 70%, 9 min, 100%; 12 min, 100%; 12.1 min, 5%. The total instrument method run time was 16 min per sample. The column chamber was at 40 °C [34], with a flow of 0.3 mL/min. The autosampler was maintained at a cool temperature of 5 °C with a small injection volume of 2 µL.

**Figure 1:**
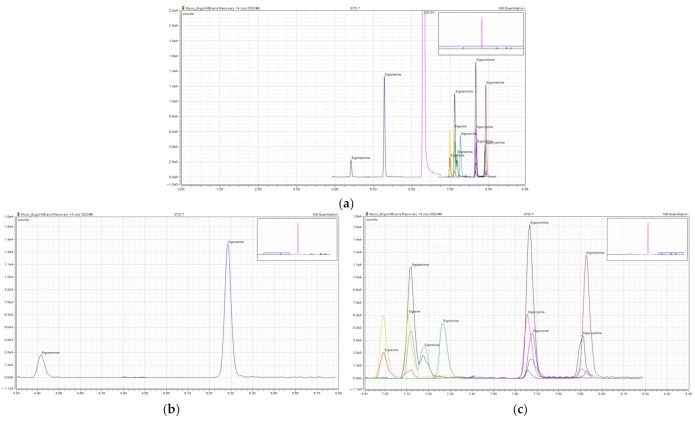
Extracted Ion Chromatograms. (**a**) Representative chromatogram of each ergot epimer analyzed at the lowest concentration on the standard curve (0.25 µg/kg) and internal standard (LSD-D_3_) at 20 µg/kg. (**b**,**c**) Zoomed versions of the chromatogram for each epimer.

The UHPLC was coupled to a ThermoFisher Scientific TSQ Altis (triple quadrapole (QqQ) tandem mass spectrometer (MS/MS)) (ThermoFisher Scientific, Waltham, MA, USA) with electrospray ionization (ESI). The mass spectrometer was used in selective reaction monitoring (SRM) mode with ESI in positive mode. Nitrogen was used as the sheath, aux, and sweep gas. Argon was used for the collision gas. The sheath gas was set at 50 Arb, aux gas at 10 Arb, sweep gas at 1 Arb and the collision induced dissociation (CID) gas at 1.5 mTorr was utilized. The ion transfer tube was set to 325 °C with the vaporizer temperature of 350 °C. The cycle time was 0.35 s. Three SRM transitions for each analyte were obtained; one qualifying ion and two qualifying/confirming ions. One precursor ion was identified following the analysis of three product ions of most common abundance. Parameters for this method are listed in Table 6. The molecular ions with a loss of water were utilized for ergosinine and ergocryptinine since they had a greater intensity compared to other precursor ions. Similar molecular ions have been used in a previous studies [23,34]. Ion identification was confirmed based on the relative ion intensity tolerances in the Commission Decision 2002/657/EC [32] with retention times within 0.6 min of the set retention time for each analyte. Data were collected using Chromeleon software (ThermoFisher Scientific, Edmonton, AB, Canada). Chromatograms for each analyte and the internal standard are shown in Figure 1.

A control wheat matrix sample containing all 12 ergot epimers and a solvent-spiked sample, with a concentration (2 µg/kg) in the mid linear range, were used as quality control samples for this analytical method to monitor performance. To measure instrument variability, each amber vial within an analytical run, containing reconstituted extract, was injected twice and analyzed. An average value for the duplicate injection was utilized.

### 5.2. Method Validation

Clean hard red spring wheat (visually free of ergot sclerotia) was analyzed to ensure the grain was free of analytes to evaluate matrix effects. Associated with low concentrations of analytes in the clean grain, the concentrations of all 12 ergot epimers were adjusted by subtracting the background concentrations [3]. This clean wheat was also used as the pseudo-blank samples (*n* = 10) for the calculation of the LOD and LOQ.

Solvent calibration curves [38,39] were used to assess linearity with the equation y = mx + b, where (y) is the peak area of analyte/peak area of internal standard and (x) is concentration. The calibration curves were weighted using ‘1/Amount’. This allows for equal weighting of the calibration points for each concentration. This is associated with the calibration curve analyzed at the beginning and end of each analytical run. Calibration curves contained seven standards for each epimer at concentrations of 0.25, 0.5, 1, 2, 10, 50, and 250 µg/kg. The dried down working standard containing all 12 ergot epimers was removed from the −80 °C freezer the day of analysis. Using methanol:water (50:50), the working standard was reconstituted in 300 µL. A series of dilutions from the working standard created the seven standard concentrations used in the calibration curve. To create the calibration curve, 160 µL of each standard concentration containing all 12 ergot epimers and 40 µL of internal standard was added to an amber autosampler vial with 200 µL spring HPLC inserts. This approach is commonly utilized [42]. The LOD and LOQ were calculated according to the Guidance Document on the Estimation of LOD and LOQ for Measurements in the Field of Contaminants in Feed and Food [32]. See Equations (1) and (2) (described in the guidance document).

(1) $$X_{LOD}\;=\;3.9\;\times \;\left(\frac{S_{y,b}}{b}\right)$$

(2) $$X_{LOQ}\;=\;3.3\;\times \;X_{LOQ}$$

*X_LOD_*: Limit of detection

*S_y,b_*: Standard deviation of pseudo-blank signals

*b*: Slope of the calibration curve at concentrations close to expected LOD

*X_LOQ_*: Limit of quantification

Matrix effects were evaluated using Equation (3) [29]. Two working standards, containing all epimers, were reconstituted in either methanol:water (50:50) (solvent matrix) or clean hard red spring wheat matrix that was extracted with acetonitrile:water (80:20), dried, and reconstituted with methanol:water (50:50) (wheat matrix). Serial dilutions from each of the working standards were conducted to obtain seven samples with concentrations of 0.25, 0.5, 1, 2, 10, 50, and 250 µg/kg for each epimer. The wheat and solvent matrix samples were then analyzed as stated previously. The analysis produced a graph of peak area of analyte (epimer) (y) vs. concentration (x) for the clean wheat matrix and solvent matrix for each epimer. The slopes from each graph for each epimer were used to calculate ME for the specific epimer. The internal standard was excluded from this calculation.

(3) $$\mathrm{ME}\;\left(\%\right)\;=\;\left(\frac{\mathrm{Slope}\;\mathrm{in}\;\mathrm{spiked}\;\mathrm{clean}\;\mathrm{wheat}}{\mathrm{Slope}\;\mathrm{in}\;\mathrm{spiked}\;\mathrm{solvent}\;}\right)\;\times \;100$$

Recovery and precision of all 12 ergot epimers were assessed by spiking all 12 epimers in ground clean wheat samples at two different concentrations, with six replicates at each concentration. The concentrations chosen were based on the linear range with a mid (5 µg/kg) concentration and a low (0.75 µg/kg) concentration. These concentrations represent the concentration spiked in the diluted samples that the instrument would detect. Availability and price of standards to spike was a limitation. Therefore, the samples were extracted as mentioned previously, however, using 1 g of ground wheat in a 25 mL plastic centrifuge tube with 4 mL of extraction solvent. The samples were diluted by a factor of 4 (4 mL extraction solvent/1 g sample).This was repeated on three separate days. Recovery was calculated as per Equation (4) [31] and averaged across all three days. Precision was examined by inter and intra-day repeatability [23]. The percent relative standard deviation (% RSD) for all six replicates on three different days was calculated to determine inter day precision for each spike concentration. Intra-day precision was determined by the % RSD from a single analysis within one day.

(4) $$\mathrm{Recovery}\;\left(\%\right)\;=\;\left(\frac{\mathrm{Concentration}\;\mathrm{measured}\;\left(µg/\mathrm{kg}\right)}{\mathrm{Concentration}\;\mathrm{spiked}\;\left(µg/\mathrm{kg}\right)}\right)\;\times \;100$$

Carry over was monitored and minimized by injecting blank samples following high epimer concentration sample analysis. A needle wash was preformed between all injections though washing the outside of the needle in a reservoir with a rinse solution containing 50:50 Acetone (Honeywell, Fisher Scientific, Edmonton, AB, Canada): water (Fisher Scientific, Edmonton, AB, Canada). Acceptable carry over was deemed when the peak area of all 12 epimers was below the lowest concentration on the calibration curve (0.25 µg/kg), in the blank sample. This method was validated following procedures from the Commission Decision 2002/657/EC [33].

### 5.3. Natural Ergot Contaminated Wheat

Six subsamples of five grams each, from each of the six independent ground and diluted ergot contaminated samples were extracted and analyzed for the concentration of 12 ergot *R* and *S*-epimers (ergotamine, ergotaminine, ergocornine, ergocorninine, ergocristine, ergocristinine, ergocryptine, ergocryptinine, ergometrine, ergometrinine, ergosine and ergosinine), using the validated method above. A dilution factor of 4 (20 mL extraction solvent/5 g ground sample) was applied to obtain the actual concentration of the epimers within the samples.

## Figures and Tables

**Table 1:** Linear equation of calibration curve, instrumental limit of detection and instrumental limit of quantification for the analysis of ergot epimers.

Epimer	Linear Equation ^a,b^	r^2 c^	S_y,b_ ^d^	b ^e^	Instrumental LOD ^f^ (µg/kg)	Instrumental LOQ ^g^ (µg/kg)
Ergometrine (*R*)	y = 2.77x + 0.0642	0.998	0.123	2.86	0.167	0.552
Ergometrinine (*S*)	y = 17.7x − 0.997	0.999	0.0379	16.6	0.00893	0.0295
Ergosinine (*S*)	y = 4.87x − 0.441	0.999	0.0449	4.41	0.0398	0.131
Ergosine (*R*)	y = 8.49x − 0.217	0.998	0.0906	8.11	0.0436	0.144
Ergotaminine (*S*)	y = 21.4x − 2.15	0.999	0.0828	5.01	0.0645	0.213
Ergotamine (*R*)	y = 6.25x − 0.633	0.999	0.118	5.50	0.0839	0.277
Ergocryptinine (*S*)	y = 5.16x − 0.436	0.998	0.201	10.4	0.0757	0.250
Ergocryptine (*R*)	y = 11.3x + 0.303	0.994	0.336	11.6	0.113	0.372
Ergocorninine (*S*)	y = 22.3x − 0.753	0.998	0.189	20.8	0.0354	0.117
Ergocornine (*R*)	y = 11.0x − 0.593	0.998	0.406	7.02	0.225	0.744
Ergocristinine (*S*)	y = 21.3x − 0.445	0.999	0.257	4.59	0.219	0.721
Ergocristine (*R*)	y = 6.92x + 0.267	0.995	0.274	19.5	0.0548	0.181

^a^ y: ratio of peak area of analyte and internal standard, ^b^ x: concentration, ^c^ r^2^: coefficient of determination, ^d^ S_y,b_: Standard deviation of pseudo-blank signals (clean wheat, *n* = 10), ^e^ b: Slope of the calibration curve at concentrations close to expected LOD, ^f^ Limit of detection (LOD) = 3.9 × S_(y,b)_/b, ^g^ Limit of quantification (LOQ) = 3.3 × LOD. These results are from a single analytical run using ultra-high performance liquid chromatography tandem mass spectrometry (UHPLC-MS/MS).

**Table 2:** Matrix effects, recovery, and inter-day precision of ergot epimers in a hard red spring wheat matrix.

Epimer	ME ^a^	Recovery ^b^		Intra-Day Precision ^c^		Inter-Day Percision ^d^	
		Low Spike (*n* = 18)	Mid Spike (*n* = 18)	Low Spike ^e^ (*n* = 6)	Mid Spike ^f^ (*n* = 6)	Low Spike (*n* = 18)	Mid Spike (*n* = 18)
	%	%	%	% RSD ^g^	% RSD	% RSD	% RSD
Ergometrine	108	109.7	119.1	3.1	12.7	23.9	22
Ergometrinine	103	115.0	118.9	2.5	12.8	18.8	19.5
Ergosinine	101	106.8	99.84	3.0	10.7	10.1	11.3
Ergosine	113	98.5	92.7	6.1	10.7	16.9	12.2
Ergotaminine	104	102.3	88.1	2.3	12.2	11.9	8.3
Ergotamine	116	87.4	74.9	3.8	7.9	9.9	9.1
Ergocryptinine	100	105.5	95.9	3.8	11.9	8.9	9.3
Ergocryptine	108	88.6	83.2	8.2	11.1	12.5	7.9
Ergocorninine	103	115.3	114.4	1.9	13.3	15.9	12.4
Ergocornine	111	95.3	85.2	3.3	9.3	14.1	9.3
Ergocristinine	106	100.6	87.1	3.1	12.5	5.9	8.0
Ergocristine	107	78.8	68.3	13.8	9.14	8.7	8.8

^a^ Matrix effects (ME)(%) = (Slope in spiked extract)/(Slope in pure solvent) × 100, ^b^ Recovery: Recovery (%) = (Concentration measured (µg/kg))/(Concentration spiked (µg/kg)) × 100 (*n* = 6 samples/concentration/3 days), ^c^ Intra-day Precision: % RSD for *n* = 6 samples on a single day, ^d^ Inter-day precision: % RSD for *n* = 6 samples on three different days (*n* = 18), ^e^ Low Spike: Clean wheat spiked at a concentration of 0.75 µg/kg, ^f^ Mid Spike: Clean wheat spiked at a concentration of 5 µg/kg, ^g^ % RSD: Relative Standard Deviation (RSD) = (Standard deviation/Mean) × 100. Ultra-high performance liquid chromatography tandem mass spectrometry was utilized for this analysis.

**Table 3:** Concentrations of each ergot alkaloid with or without the internal standard, deuterated lysergic acid diethylamide (LSD-D_3_).

	Low Spike ^a^		Mid Spike ^b^	
	Theoretical (µg/kg)	Measured with Internal Standard (µg/kg)	Theoretical (µg/kg)	Measured with Internal Standard (µg/kg)
Ergocornine	0.75	0.80	5	4.48
Ergocorninine	0.75	0.94	5	5.99
Ergocristine	0.75	0.71	5	3.66
Ergocristinine	0.75	0.91	5	4.66
Ergocryptine	0.75	0.73	5	4.38
Ergocryptinine	0.75	0.92	5	5.08
Ergometrine	0.75	0.92	5	6.18
Ergometrinine	0.75	0.85	5	6.16
Ergosine	0.75	0.80	5	4.84
Ergosinine	0.75	0.89	5	5.25
Ergotamine	0.75	0.77	5	3.98
Ergotaminine	0.75	0.79	5	4.58

^a^ Low Spike: Clean wheat spiked at a concentration of 0.75 µg/kg, ^b^ Mid Spike: Clean wheat spiked at a concentration of 5 µg/kg. Values are expressed as the mean (*n* = 18).

**Table 4:** Total, *R*, and *S*-epimer concentrations from natural ergot contaminated hard red spring wheat.

	Total Concentration of Epimers (µg/kg)	Total *R*-Epimer Concentration (µg/kg)	Total *S*-Epimer Concentration (µg/kg)	% of *R*-Epimer	% of *S*-Epimer
Mean ^a^ ± SD ^b^	841 ± 92	544 ± 79	290 ± 50	65 ± 5	35 ± 5

^a^ Mean concentration (µg/kg) (*n* = 6), ^b^ SD: Standard deviation. Samples were analyzed by the validated UHPLC-MS/MS method.

**Table 5:** Concentration of each epimer analyzed in natural ergot contaminated hard red spring wheat samples.

	Independent Sample Number											
	1		2		3		4		5		6	
	Mean ^a^	SD ^b^	Mean	SD	Mean	SD	Mean	SD	Mean	SD	Mean	SD
Ergocornine	52.0	6.5	52.1	11.7	61.1	6.5	8.4	2.2	58.4	8.4	61.5	19.8
Ergocorninine	42.4	5.3	37.5	8.8	35.7	5.1	6.3	1.6	38.6	6.8	58.7	14.6
Ergocristine	213.5	23.7	238.6	68.5	301.8	36.3	471.2	34.9	235.7	28.1	252.3	27.1
Ergocristinine	148.9	11.6	122.4	35.4	130.8	21.9	169.8	15.1	125.9	12.0	181.1	16.4
Ergocryptine	83.0	14.7	100.7	17.4	115.2	10.6	54.4	16.9	94.1	13.1	92.0	12.4
Ergocryptinine	56.5	8.7	56.8	11.6	54.3	7.2	26.7	6.1	49.1	7.7	78.0	5.8
Ergometrinine	10.2	2.1	7.2	2.2	7.4	2.3	5.9	0.9	8.5	1.3	11.2	1.6
Ergometrine	22.5	4.0	15.8	4.8	19.6	5.8	20.6	3.0	21.0	2.9	27.2	4.4
Ergosine	21.8	3.0	23.1	5.6	31.8	2.8	10.8	3.0	26.6	2.9	30.5	4.6
Ergosinine	12.0	1.3	12.0	3.8	14.6	1.0	6.4	1.7	12.3	1.2	17.3	2.4
Ergotamine	63.2	7.5	64.0	18.2	86.8	9.0	113.1	15.6	68.0	9.9	85.4	9.0
Ergotaminine	30.4	3.3	26.0	7.6	30.7	4.6	48.5	8.5	26.9	3.0	41.7	4.7

^a^ Mean (*n* = 6) Concentration (µg/kg), ^b^ SD: Standard deviation. Samples were analyzed by the validated UHPLC-MS/MS method.

**Table 6:** Mass spectrometry parameters set for the detection and quantification of 12 ergot epimer analytes and internal standard including retention time, precursor ion, products ions, molecular ion, radio frequency lens, and collision energy.

Epimer	Retention Time (min)	Precursor Ion (*m*/*z* ^a^)	Molecular Ion ^b,c^	RF ^c^ Lens (V)	Product Ion (*m*/*z*) (Quantifier (Q))	Product Ion (*m*/*z*) (Qualifier (C))	Collision Energy (eV) (Q/C)
Ergometrine	4.41	326.14	[M+H]^+^	82	282.96	179.97, 197.08	16.92/34.91, 22.02
Ergometrinine	5.29	326.14	[M+H]^+^	82	208	222.99, 282.16	24.5/27.49, 18.77
Ergosinine	6.98	530.3	[M-H_2_O+H]^+^	84	223.16	263.16, 277.05	28.73/27.67, 22.36
Ergosine	7.10	548.3	[M+H]^+^	84	223.08	208.07, 268.16	33.62/40.82, 24.52
Ergotaminine	7.11	582.21	[M+H]^+^	73	564.05	223.03, 29.03	13.64/31.62, 27.28
Ergotamine	7.17	582.21	[M+H]^+^	73	223.03	564.10, 207.96	32.13/14.39, 41.35
Ergocryptinine	7.87	558.3	[M-H_2_O+H]^+^	109	305	291.08, 348.14	24.18/22.97, 19.82
Ergocryptine	7.63	576.3	[M+H]^+^	109	268.16	208.07, 223.08	25.77/44.23, 36.84
Ergocorninine	7.65	562.3	[M+H]^+^	79	544.25	277.14, 305.05	15.76/28.16, 27.67
Ergocornine	7.26	562.3	[M+H]^+^	79	268.14	208.07, 223.08	25.2/42.91, 36.23
Ergocristinine	7.89	610.3	[M+H]^+^	109	592.29	305.05, 325.08	14.89/28.88, 27.25
Ergocristine	7.66	610.3	[M+H]^+^	109	268.16	208.08, 348.10	26.23/43.66, 25.62
LSD-D_3_ ^d^	6.27	327.3	[M+H]^+^	75	226.21	n/a	24.46

^a^ *m*/*z*: mass/charge, ^b,c^ [M+H]^+^: Analyte plus a hydrogen, positively charged. [M-H_2_O+H]^+^: Analyte minus a water molecule plus a hydrogen, positively charged, ^c^ RF:Radio frequency, ^d^ LSD-D_3_: Deuterated lysergic acid diethylamide (internal standard).

## Data Availability

The data presented in this study are available through the corresponding author.
